# Unraveling the electrochemical and spectroscopic properties of neutral and negatively charged perylene tetraethylesters

**DOI:** 10.1038/s41598-021-95551-0

**Published:** 2021-08-09

**Authors:** Christian Wiebeler, Joachim Vollbrecht, Adam Neuba, Heinz-Siegfried Kitzerow, Stefan Schumacher

**Affiliations:** 1 Institut für Analytische Chemie, Leipzig University, 04103 Leipzig, Germany; 2 Wilhelm-Ostwald-Institut für Physikalische und Theoretische Chemie, Leipzig University, 04103 Leipzig, Germany; 3 Leibniz Institute of Surface Engineering, 04318 Leipzig, Germany; 4 Department of Chemistry, University of Paderborn, 33098 Paderborn, Germany; 5 Center for Optoelectronics and Photonics Paderborn, University of Paderborn, 33098 Paderborn, Germany; 6 Department of Physics, University of Paderborn, 33098 Paderborn, Germany; 7 Present Address: Institute of Physics and Astronomy, University of Potsdam, 14476 Potsdam-Golm, Germany

**Keywords:** Chemistry, Materials science, Physics

## Abstract

A detailed investigation of the energy levels of perylene-3,4,9,10-tetracarboxylic tetraethylester as a representative compound for the whole family of perylene esters was performed. It was revealed via electrochemical measurements that one oxidation and two reductions take place. The bandgaps determined via the electrochemical approach are in good agreement with the optical bandgap obtained from the absorption spectra via a Tauc plot. In addition, absorption spectra in dependence of the electrochemical potential were the basis for extensive quantum-chemical calculations of the neutral, monoanionic, and dianionic molecules. For this purpose, calculations based on density functional theory were compared with post-Hartree–Fock methods and the CAM-B3LYP functional proved to be the most reliable choice for the calculation of absorption spectra. Furthermore, spectral features found experimentally could be reproduced with vibronic calculations and allowed to understand their origins. In particular, the two lowest energy absorption bands of the anion are not caused by absorption of two distinct electronic states, which might have been expected from vertical excitation calculations, but both states exhibit a strong vibronic progression resulting in contributions to both bands.

## Introduction

During the recent decades, considerable improvements in organic electronics have been achieved, resulting in the commercialization of organic light emitting diodes (OLEDs) as competitive display technology^1^ and potentially as an option for general lighting^2^. While not as prominent and widespread as OLEDs, affordable and flexible circuitry based on organic field effect transistors^3–8^, energy production via photovoltaics^9–12^, thermoelectrics^13,14^, or the ratchet effect^15^, sensors^16^ and photodetectors^17–19^, as well as applications in biotechnology^20–22^ have also experienced steady advances. The pacemaker for the aforementioned improvements were mainly the synthesis and investigation of new organic semiconductor compounds^23–29^. For instance, the expansive family of perylene diimides (PDIs) has been investigated extensively, both fundamentally and as crucial component in various applications^30–35^. In contrast, the less known perylene tetraesters (PEs) and their derivatives have not yet received comparable attention, although several studies can be found in the literature that focus on their fundamental electronic, spectroscopic, and mesogenic properties^36–40^, as well as on their applicability as emitter material in OLEDs^41–45^ or as building block for electron acceptor polymers in organic solar cells^46^. Hence, a firm understanding of the reduced species of perylene imides and esters is necessary, since both types of compounds are either commonly used as electron transporting layers (PDIs) or electron acceptors or have the potential of becoming widely used in the future (PEs), respectively. In the case of PDIs, studies have been performed recently on the reduced states of several imide derivatives^47–49^. However, to the best of our knowledge similar investigations into their ester counterparts have not yet been reported.

This observation appears to also hold for quantum chemical studies of charged perylene esters. In case of the imides, results for the spectroscopic properties of the monoanionic and dianionic species from such calculations have been published recently^48,49^. These theoretical investigations are based on density functional theory (DFT) and mainly employed the B3LYP functional with an exception being the calculation of interconversion energies, for which the PBE0 functional was used. In contrast to this, DFT-based computational studies of perylene esters have mainly reported ground state properties of neutral molecules^45,46,50^ and only the latter has also considered an anion^50^. Based on our previous simulations of related neutral molecules^36–38,43^, we extend our modelling to reduced perylene esters. For this purpose, we benchmark the results from DFT-based calculations for a selection of functionals with the post-Hartree–Fock methods RI-CC2 and RI-ADC(2) and analyze the nature of absorption bands by the inclusion of vibronic effects.

Hence, in this study the electrochemical and spectroscopic properties of perylene-3,4,9,10-tetracarboxylic tetraethylester (PTTE) as a representative compound for the whole family of perylene esters is investigated and special attention is given to its two types of reduced species, namely the monoanion (PTTE$^{-}$) and the dianion (PTTE$^{2-}$). The experimental results are flanked by an assessment of quantum chemical methods for the calculation of excited states for the neutral and reduced species. Based on this, we are able to understand the origins of the absorption bands in the visible region and their changes induced by reduction. Furthermore, the molecular orbitals that are involved in the relevant electronic transitions are discussed and the impact of vibronic effects is investigated.

## Results

### Experiments

The first experiments that were conducted in this study were cyclovoltammetry (CV) and optical absorption spectroscopy of PTTE in solution. While these basic measurements have been previously reported^37^, it is important to ascertain the spectroscopic properties of the neutral PTTE and to establish the electrochemical potentials required to oxidize or reduce the neutral PTTE. A precise knowledge of these potentials is paramount before any more advanced measurements can take place.

**Figure 1:**
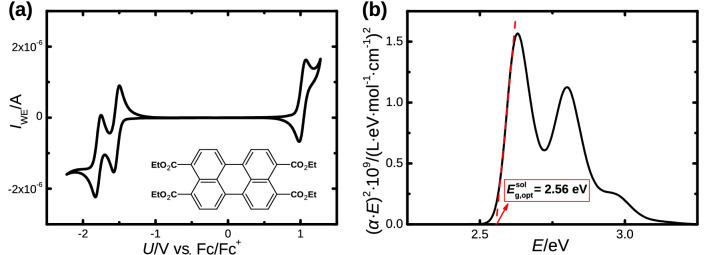
(**a**) Cyclic voltammogram of PTTE solved in CH$_2$Cl$_2$ referenced to the internal standard Fc/Fc$^+$. The inset shows the chemical structure of PTTE. (**b**) Tauc plot of PTTE in solution under the assumption of a direct allowed transition. The tangent of the low energy edge of the Tauc plot (red dashes) yields the value for the optical bandgap when it crosses the abscissa ($E^{\text {sol}}_{\text {g,opt}} = 2.56$ eV).

As can be seen from the CV measurements in Fig. 1a, PTTE shows two well resolved reduction and one oxidization potential. In contrast to the electrochemical investigation of most organic semiconductors, the simultaneous electrochemical determination of the first reduction and first oxidation potential of PTTE allows the calculation of the bandgap purely by electrochemical means (cf. Table 1). In most other cases, a combination of the optical bandgap and one electrochemical potential, either reduction or oxidation, is used to map out the relevant energy levels, namely the highest occupied molecular orbital (HOMO) and the lowest unoccupied molecular orbital (LUMO). The following equation was used to determine the HOMO and LUMO energy in solution from the measured potentials:

1 $$\begin{aligned} E^{\text {sol}}_{{\text {HOMO,LUMO}}}= -eU_{\text {ox,red}} + (-\,4.44\,{\text {eV}} - 0.46\,{\text {eV}}), \end{aligned}$$

where $U_{\text {ox,red}}$ is the oxidation or reduction potential, respectively, *e* is the elementary charge, and where the reference potential of the NHE (- 4.44 eV) and the potential of the internal standard Fc/Fc$^+$ in CH$_2$Cl$_2$ (− 0.46 eV) were taken into account^51^. Furthermore, it has been shown that the addition of empirical corrections to Eq. (1) yield good estimates of the HOMO and LUMO energy for crystalline or molecular solids^52^. In the case for the HOMO energy in a solid, the following equation was used:

2 $$\begin{aligned} E^{\text {c}}_{\text {{HOMO}}}= -\alpha ^+ \cdot eU_{\text {ox}} + \beta ^+, \end{aligned}$$

where $\alpha ^+$ and $\beta ^+$ are empirical correction factors for the oxidation. The exact values for polycyclic aromatic hydrocarbons are $\alpha ^+ = 1.47$ and $\beta ^+ = -\,4.26\,{\text {eV}}$, respectively. In the case for the LUMO energy in a solid, the following equation was used:

3 $$\begin{aligned} E^{\text {c}}_{{\text {LUMO}}}= -\alpha ^- \cdot eU_{\text {red}} + \beta ^-, \end{aligned}$$

where $\alpha ^-$ and $\beta ^-$ are empirical correction factors for the reduction. The exact values for polycyclic aromatic hydrocarbons are $\alpha ^- = 1.07$ and $\beta ^- = -\,4.45\,\text {eV}$, respectively^52^. The results using the two different methods mentioned above yield electrochemical bandgaps for the solution of $E^{\text {sol}}_{\text {g,ec}} = 2.5\pm 0.1\,{\text {eV}}$ and for the molecular solid of $E^{\text {c}}_{\text {g,ec}} = 3.0\pm 0.1\,{\text {eV}}$ (cf. Table 1), a difference that is not uncommon^52^. Furthermore, the LUMO energies are shifted upward in the solid, whereas the HOMO energy remains comparable to the solution value.

Nonetheless, the optical bandgap of PTTE was still determined, since it can act as a useful verification of the electrochemically measured bandgap. To this end a Tauc plot was used under the assumption of an allowed, direct transition, which results in an ordinate of $\left( \alpha \cdot E \right) ^2$, where $\alpha$ is the absorption coefficient and *E* is the photon energy, to obtain the optical bandgap. The tangent of the low energy edge of the Tauc plot can then be extrapolated to the point where it crosses the abscissa, which should be equal to the optical bandgap (cf. Fig. 1b)^53–55^. The bandgap obtained purely from the electrochemical potentials for the solution ($E^{\text {sol}}_{\text {g,ec}} = 2.5\pm 0.1\,{\text {eV}}$), matches the optical bandgap determined via the Tauc plot ($E_{\text {g,opt}} = 2.56\,{\text {eV}}$) quite well. More details regarding the CV measurements can be found in the literature^37^.

**Table 1:** Reduction and oxidation potentials of PTTE solved in CH$_2$Cl$_2$ referenced to the internal standard Fc/Fc$^+$, with [N(n-Bu)$_4$]PF$_6$ as supporting electrolyte, a Pt working electrode, and at a scan rate of $v = 50\,{\text {V/s}}$.

	Ox/HOMO	Red$_1$/LUMO	Red$_2$/LUMO+1	$E_{\text {g,ec}}$/eV
*U*/V	1.0	− 1.5	− 1.8	
$E^{\text {sol}}$/eV	− 5.9 ± 0.1	− 3.4 ± 0.1	− 3.1 ± 0.1	2.5 ± 0.1
$E^{\text {c}}$/eV	− 5.8 ± 0.1	− 2.8 ± 0.1	− 2.5 ± 0.1	3.0 ± 0.1

**Figure 2:**
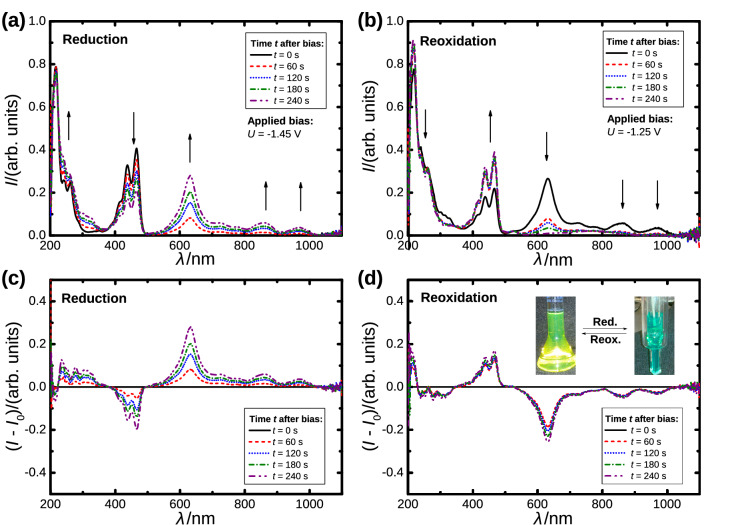
(**a**) Time dependent absorption spectra during reduction of PTTE under an applied bias of $U = -\,1.45$ V. (**b**) Time dependent absorption spectra during reoxidation of the reduced PTTE under an applied bias of $U = -\,1.25$ V. (**c**,**d**) Relative changes in the time dependent absorption spectra during reduction and reoxidation of PTTE. The spectra at the time of applying the bias *U* were selected as reference intensity $I_0$. The inset in (**d**) exhibits the changes in color during reduction and reoxidation.

The next natural step in the investigation of the reduced species of PTTE is to measure the spectroscopic properties. Here we refrain from further investigating the oxidized species (PTTE$^{+}$) that was also reasonably well resolved in the CV measurements, as the application of PTTE is mostly related to being an electron acceptor. Time dependent spectroelectrochemical measurements were performed to obtain the absorption spectra of the reduced species and the applied potentials were chosen in such a way to allow a reduction and a complete reoxidation back to the neutral PTTE ($U_{\text {red}}=-\,1.45\,{\text {V}}$; $U_{\text {reox}}=-\,1.25\,{\text {V}}$). The reduced species can readily be distinguished from the neutral PTTE by the naked eye, as the neutral solution loses its yellow color once the reduction sets in and the solution turns turquoise (cf. Fig. 2). The color perception of the solution changes since the absorption band in the region of $\lambda = 400{-}500\,{\text {nm}}$ weakens in intensity while at the same time several bands at longer wavelengths rise in intensity. The most prominent increase can be seen in the region of $\lambda = 600{-}650\,{\text {nm}}$, whereas it is possible to discern less pronounced absorption bands at $\lambda \approx 850\,{\text {nm}}$ and $\lambda \approx 950\,{\text {nm}}$ that were not present in the spectra of the neutral compound.

### Quantum chemical calculations

#### Benchmark for vertical excitations

One concern in DFT-based calculations is the choice of an appropriate functional. To address this issue, we decided to employ two post-Hartree–Fock methods as reference: RI-CC2 and RI-ADC(2). The excitation energies and oscillator strengths obtained from the conventional methods are reported in Table 2, whereas the results for the spin-scaled variants of RI-CC2 and -ADC(2) are discussed in the ESI, see also Table S1 and Table S2. For both methods, the lowest-lying excited state of the neutral molecule is bright with an oscillator strength slightly above $0.60$. Furthermore, their energies differ by $0.03 \, {\mathrm {eV}}$ only and with a wavelength of around $455 \, {\mathrm {nm}}$ these transitions are located in the region of the experimental absorption band.

**Table 2:** Excitation energies for states with oscillator strengths larger than 0.02 for the neutral, monoanionic, and dianionic species of PTTE obtained via RI-CC2 or -ADC(2) with aug-cc-pVDZ basis set based on MP2/6-311G(d,p) optimized structures.

Exp.		CC2			ADC(2)		
$\Delta$E (eV)	$\lambda$ (nm)	$\Delta$E (eV)	$\lambda$ (nm)	f	$\Delta$E (eV)	$\lambda$ (nm)	f
**Neutral**							
2.76	450	2.74	452	0.6086	2.71	457	0.6426
**Anion**							
1.28	970	1.57	791	0.0611	1.50	824	0.0594
1.44	860	1.59	778	0.1274	1.55	799	0.1811
1.97	630	2.29	541	0.5984	2.31	537	0.4645
**Dianion**							
–	–	1.76	704	0.1746	1.69	733	0.2018
–	–	2.43	510	0.8300	2.38	520	0.7760

For the monoanion, both methods yield two relatively dark low-lying states in the range of $1.50 \, {\mathrm {eV}}$ to $1.60 \, {\mathrm {eV}}$, which might be the origin of the absorption in the infrared part of the spectrum found experimentally. Interestingly, the difference in excitation energy is larger for RI-ADC(2) than for RI-CC2, but in both cases it is somewhat smaller than the difference between the experimental absorption maxima. Another absorption feature that is connected to the formation of the monoanionic species is located around $630 \, {\mathrm {nm}}$. This is caused by a bright state with similar oscillator strength as the bright state of the neutral molecule and it is the fourth excited state in the RI-CC2 calculation, whereas it is the third one in case of RI-ADC(2). For both methods, the corresponding excitation energies are more than $0.30 \, {\mathrm {eV}}$ higher than the experimental absorption feature resulting in the largest deviation.

Finally, the calculations predict that the dianion will exhibit absorption at two distinguished positions: Slightly higher in energy than the two lowest-lying states of the monoanion and in-between the prominent absorption bands of the neutral and the singly charged species. For both methods and in both regions, an excited state with an oscillator strength larger than 0.10 is found, but there are also several nearly dark states close to them. Therefore, the mentioned states are the fifth and eleventh for RI-CC2 and the fourth and eleventh for RI-AD(2). Overall, with increasing negative charge of the molecule more and more excited states have to be determined to cover a certain spectral range. Furthermore, the agreement between the two methods is rather high with a maximum deviation in excitation energy of ca. $0.07 \, {\mathrm {eV}}$ and a comparable ordering of the excited states.

**Table 3:** Excitation energies for states with oscillator strengths larger than 0.02 for the neutral, monoanionic, and dianionic species of PTTE obtained via regular TD-DFT/aug-cc-pVDZ calculations employing the functionals PBE0 or CAM-B3LYP (CAM) on MP2/6-311G(d,p) optimized structures.

Exp.		PBE0			CAM		
$\Delta$E (eV)	$\lambda$ (nm)	$\Delta$E (eV)	$\lambda$ (nm)	f	$\Delta$E (eV)	$\lambda$ (nm)	f
**Neutral**							
2.76	450	2.53	490	0.5261	2.76	449	0.6189
**Anion**							
1.28	970	1.52	817	0.0448	1.64	757	0.0591
1.44	860	1.58	784	0.0785	1.66	748	0.1315
1.97	630	2.24	554	0.5215	2.27	545	0.5395
**Dianion**							
–	–	1.72	721	0.1203	1.90	652	0.1722
–	–	2.45	506	0.6167	2.61	475	0.7990

As shown in Table S3 and Table S4 of the ESI, the excitation energies are rising with increasing amount of exact exchange. Therefore, PBE0 exhibits the highest excitation energies among the global hybrid functionals and the results are also given in Table 3. In particular, the first two reported excitation energies of the monoanion and the first of the dianion lie in-between their counterparts from the conventional post-Hartree–Fock calculations. Relative to RI-CC2, the excitation energy of the third bright state for the monoanion is underestimated by $0.05 \, {\mathrm {eV}}$ and that of the second bright state for the dianion is overestimated by $0.02 \, {\mathrm {eV}}$. In contrast to this, the excitation energy of the first excited state for the neutral molecule is $0.17 \, {\mathrm {eV}}$ smaller than from RI-ADC(2). In addition, the state ordering with this functional is the same as for RI-CC2 with the only exception being the second reported state of the dianion, which is the third excited state for PBE0, but the fourth for RI-CC2. Altogether, the $25\%$ of exact exchange in the global hybrid functional PBE0 appear as a sensible choice, as further increasing this amount is expected to increase the agreement for the first excited state of the neutral molecule relative to the post-Hartree–Fock methods, but it is also to be expected that it worsens the results for the charged species.

As an alternative, a long-range separated functional like CAM-B3LYP with a variable amount of exact exchange might be employed. The results from these calculations are also shown in Table 3. Regarding the state ordering, for the dianion the first and seventh excited state are bright, whereas the first excited state of the neutral molecule and the first three excited states of the monoanion are bright again. Therefore, the doubly charged species exhibits fewer dark states with this functional relative to RI-ADC(2), but the same ordering is found for the other two species. For the excitation energies, most of them are overestimated, in particular in case of the charged molecules. The only exception is the excitation energy of the third excited state from the monoanion, which is still $0.02 \, {\mathrm {eV}}$ too low. Overall and relative to the post-Hartree–Fock calculations, the brightest state of each species is best described with the CAM-B3LYP functional, whereas PBE0 yields better agreement for the energies of the darker states. Therefore, CAM-B3LYP appears to be the more reasonable choice for spectrum calculation and wavefunction analysis. We also assessed the influence of the solvent on absorption by employing the polarizable continuum model (PCM) with dichloromethane as solvent in combination with the CAM-B3LYP functional, see Table S4. This leads to red shifts of the absorption energies and it more strongly affects the states with the higher oscillator strengths. In addition, the separation of the two relatively dark states of the monoanion slightly increases. Nonetheless, the inclusion of PCM does not change the results qualitatively.

In addition to the regular time-dependent DFT (TD-DFT) calculations, we also realized calculations with the semiempirical simplified TD-DFT (sTD-DFT) method employing the same functionals as before, see Table S5 and Table S6 of the ESI. Overall, the performance is not as systematic as for the ab initio methods, but the qualitative trends are recovered: The neutral form exhibits the highest excitation energy for the states of interest; for the monoanion, there are two weaker transitions in the near-IR to red region and one stronger transition, but the latter is only slightly red-shifted relative to the transition of the neutral form; the dianion exhibits one weak transition in the near-IR and one stronger transition in the visible range, but the former lies now between the two weak transitions of the monoanion and the latter is slightly red-shifted relative to the brightest monoanion transition. With this semiempirical approach, all excitation energies from calculations with PBE0 are too small, whereas this is ameliorated with CAM-B3LYP. Given the good performance of the range-separated functional also for regular TD-DFT calculations, it appears also as most viable choice for sTD-DFT, but one should be aware of its shortcomings, in particular for the dianion.

#### Spectra of the individual species and characterization of electronic transitions

**Figure 3:**
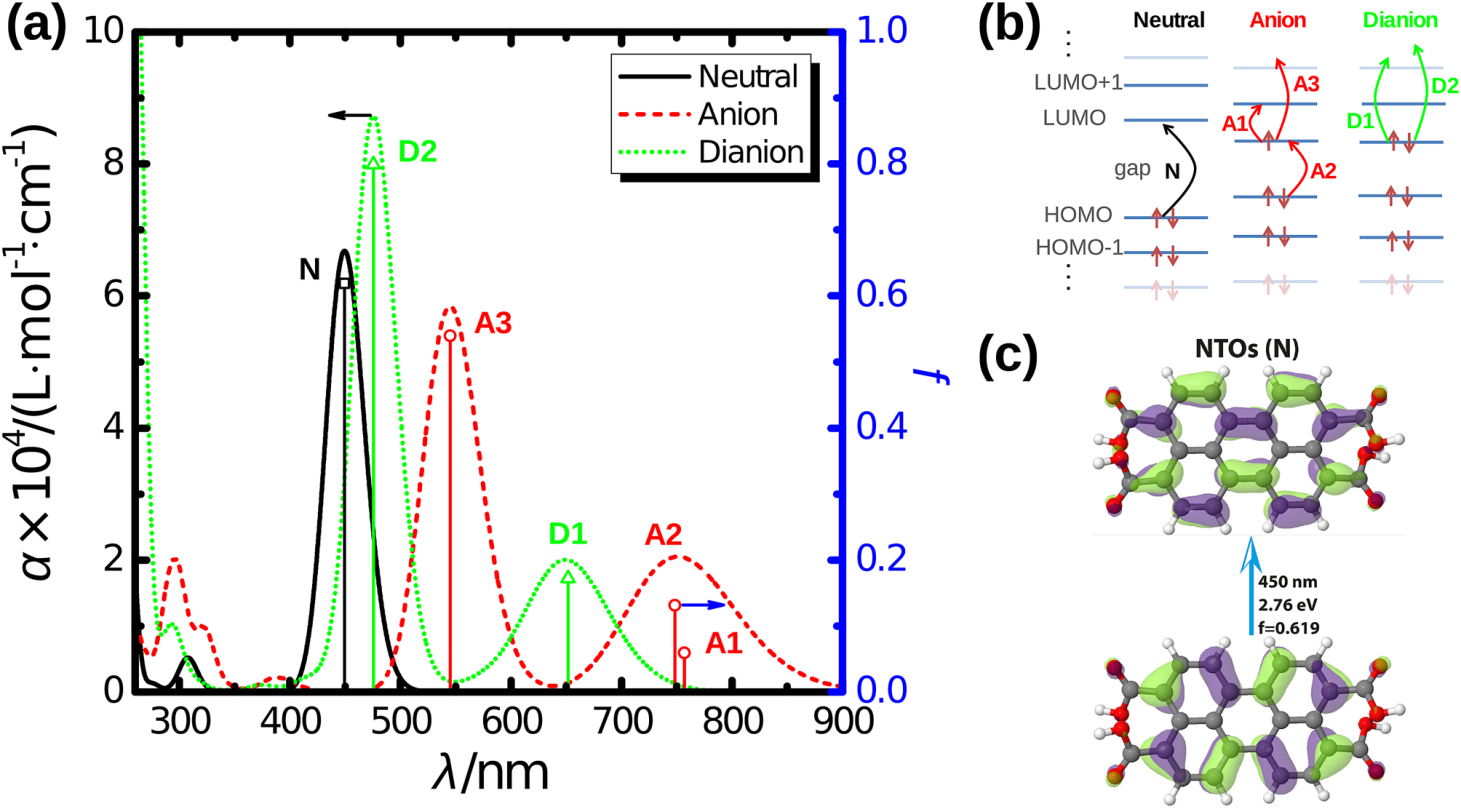
(**a**) Absorption spectra of the neutral (black) molecule as well as of the anionic (red) and dianionic (green) species. The absorption coefficient $\alpha$ (continuous graphs) and the oscillator strength *f* (open symbols with drop-lines) of the different species are depicted on the left and right ordinate, respectively. (**b**) Scheme illustrating the molecular orbitals that are involved in the electronic transitions responsible for absorption. (**c**) Natural transition orbitals (NTO) for the first excited state of the neutral molecule corresponding to an excitation from the highest occupied molecular orbital (HOMO) to the lowest unoccupied molecular orbital (LUMO). The excited state calculations employed CAM-B3LYP/aug-cc-pVDZ for MP2/6-311G(d,p) optimized structures.

To more directly compare the results from our excited state calculations with experiments, broadened spectra based on CAM-B3LYP calculations are shown in Fig. 3a. The lowest energy absorption peak of the neutral molecule (N) is located around $450 \, {\mathrm {nm}}$. The absorption in this region is caused only by the ${\mathrm {S}}_{1}$ state, as the ${\mathrm {S}}_{2}$ state is found at around $330 \, {\mathrm {nm}}$. Therefore, the shape of the absorption band in experiment is caused by a series of vibronic transitions, which could be proven theoretically with the simulation of vibronically resolved spectra for this transition, *vide infra*.

Upon addition of an electron, new absorption bands appear at around $750 \, {\mathrm {nm}}$ and $550 \, {\mathrm {nm}}$. The two lowest energy transitions are found slightly above (A1) and below (A2) $750 \, {\mathrm {nm}}$. Although the difference in their energies is too small, they might be related to the two experimentally found absorption peaks, which rise in intensity over time but remain relatively weak. In contrast to this, the absorption peak close to $550 \, {\mathrm {nm}}$ (A3) exhibits a much higher absorption intensity. This peak is about $100 \, {\mathrm {nm}}$ red shifted to the main peak of the neutral molecule. A similar spectral feature, but at around $630 \, {\mathrm {nm}}$ is also observed experimentally. Furthermore, no electronic transition exists in the spectral region, where the neutral molecule exhibits its first absorption band. Therefore, the formation of the monoanion leads to lower absorption in this part. Finally, we also observe an increase in absorption around $300 \, {\mathrm {nm}}$ when going from the neutral molecule to the monoanion. Overall, most of the changes in absorption during reduction can be traced back to the formation of the monoanion.

For the dianion, we expect an additional absorption slightly below $650 \, {\mathrm {nm}}$ (D1), i.e. between the first two absorption bands of the monoanion. Furthermore, a second absorption band (D2) is found close to the original absorption of the neutral molecule and also its intensity is comparable. Overall, the dianionic species can not be as safely identified as the monoanionic form, but it might be also present in experiment, as the absorption between $700 \, {\mathrm {nm}}$ and $800 \, {\mathrm {nm}}$ is also rising. In addition, our quantum chemical calculations give insights into the nature of the excitations underlying the absorption peaks. For this purpose, the molecular orbitals involved in the excitations can be analyzed, see Fig. 3b. In case of the neutral molecule, the first excited state is of interest, which is responsible for the absorption band between $400\, {\mathrm {nm}}$ and $450 \, {\mathrm {nm}}$. This transition is dominated by an excitation from the HOMO to the LUMO. For the monoanion, three excited electronic states are of particular interest, as they are resulting in additional absorption bands that are red-shifted relative to the neutral molecule. A1 and A2 are found around $750 \, {\mathrm {nm}}$. The former is dominated by a transition from the now singly occupied molecular orbital (SOMO) to the next higher MO, whereas the latter is caused by a transition to the SOMO from the next lower MO. A3, which is the origin of the absorption around $620 \, {\mathrm {nm}}$, is again an excitation from the SOMO, but now to higher lying MOs. In case of the dianion, this orbital is now doubly occupied and the transitions responsible for absorption start from there, but go into different higher lying unoccupied MOs.

For transitions N, A1, and A2, one pair of canonical molecular orbitals is sufficient to describe them, but more pairs are needed for A3, D1 and D2. To alleviate this, natural transition orbitals (NTOs) can be determined yielding a minimum number of single-particle orbitals to describe each electronic transition of interest^56^. The resulting NTOs for the neutral molecule are shown in Fig. 3c and correspond to a transition from HOMO to LUMO. The NTOs for the charged species can be found in Fig. S1 of the ESI. For the neutral molecule and the dianion, each of the transitions is well described by one pair with a weight of more than $95\%$, which also holds for A1. However, this weight is somewhat smaller for A2 and A3, but it is around $87\%$ nonetheless. Therefore, one pair of NTOs for each transition can be employed to illustrate the nature of the excited state of interest.

#### Structure of selected absorption bands

**Figure 4:**
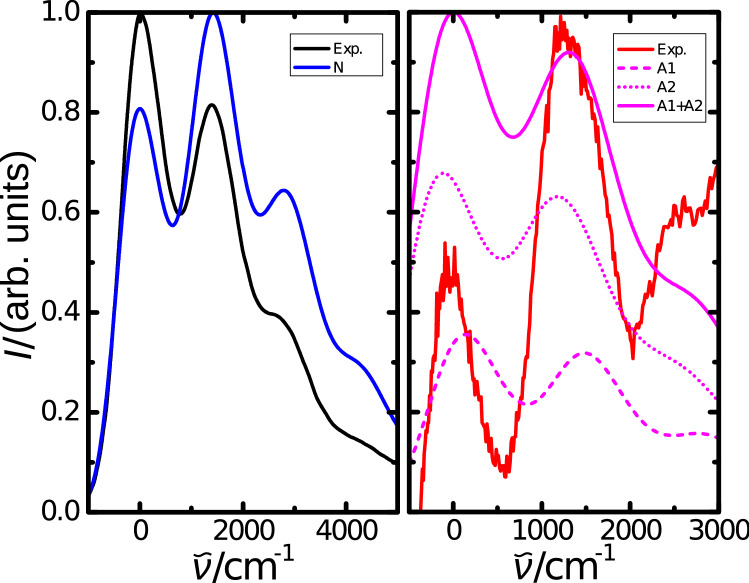
Lowest energy absorption band of the neutral molecule and simulated vibronically-resolved absorption spectrum for N. The energies, by which the spectra are shifted, were set to $21{,}412 \, {\mathrm {cm}}^{-1}$ and $21{,}350 \, {\mathrm {cm}}^{-1}$ for the experimental and simulated spectra, respectively. Absorption in the infrared region found experimentally and simulated vibronically-resolved spectra for states A1 and A2. The energies, by which the spectra are shifted, were set to $10{,}360 \, {\mathrm {cm}}^{-1}$ and $11{,}860 \, {\mathrm {cm}}^{-1}$ for the experimental and simulated spectra, respectively.

The obtained vertical absorption spectra allow to understand most of the spectroscopic features found experimentally. However, the structure of the lowest energy absorption band is not reproduced and the anion does not exhibit two well separated peaks in the region above $700 \, {\mathrm {nm}}$ in our simulations. Possible origin of these additional features might be changes in the conformation of the conjugated system, but perylene is rather rigid and the alkyl side chains are not involved in the electronic transitions. Owing to this, different conformations appear to be unlikely. In addition, there are no further electronic states in the spectral regions of interest than N in the region between $400$ and $500 \, {\mathrm {nm}}$ as well as A1 and A2 in the spectral range from $700$ to $900 \, {\mathrm {nm}}$. Another explanation might be aggregation, but in experiments no dependence of the absorption band shape on the solvent or concentration could be found. Instead, even in thin films the absorption band shape is rather similar^37^.

Therefore, vibronic effects appear to be the most promising explanation for the additional spectroscopic features. These effects are not accounted for in the discussed vertical excitation calculations and they were already identified as the origin of the structure in the lowest energy absorption band for the neutral molecule, see Fig. 4 and our previous publication^36^. To further investigate this, simulations of vibronically resolved spectra were realized with the less diffuse basis set 6-311+g(d,p), which nonetheless results in similar spectra as calculations with the aug-cc-pVDZ basis, see Fig. S2 of the ESI. Furthermore, we will focus on the structure of the absorption bands, but not their positions, which are shown in Fig. S3 of the ESI and discussed there.

Besides the pronounced vibronic progression of the neutral molecule, another remarkable feature of the experimental spectra are the two peaks in the near-IR. Based on the results for vertical excitations, we would not expect two so well separated peaks, as the energies of the two transitions are quite similar. Therefore, we decided to investigate vibronic effects for A1 and A2. The resulting spectra are also shown in Fig. 4. Interestingly, both electronic transitions result in 0-0 energies close to each other and exhibit a pronounced vibronic structure. Summing up the absorption of both states leads to a similar absorption band as found experimentally. Therefore, the absorption in this region is not made-up of two electronic transitions leading to two well separated absorption peaks, but both states contribute to both peaks. Furthermore, the excitation from the HOMO to the SOMO (A2) leads to stronger absorption than the excitation from the SOMO to the LUMO (A1). We also note that for all three optical transitions, the vibrational modes contributing the strongest to the vibronic progression are quite similar, which is discussed in more detail at the end of the ESI, see Figs. S4–S7.

## Conclusions

In conclusion the relevant energy levels of the PTTE molecule in neutral, reduced, and oxidized form were investigated in detail. Electrochemical measurements revealed one oxidation and two reduction potentials from which the eletrochemical bandgap could be determined for solutions and for solids. In the case of solutions, the electrochemical bandgap ($E^{\text {sol}}_{\text {g,ec}} = 2.5\pm 0.1\,{\text {eV}}$) matches well with the optical bandgap obtained via absorption spectroscopy and a Tauc plot analysis of solutions ($E^{\text {sol}}_{\text {g,opt}} = 2.56\,{\text {eV}}$). Furthermore, the changes of the absorption spectra were determined in dependence of the applied electrochemical potential, allowing the investigation of the two reduced species, the monoanion PTTE$^{-}$ and dianion PTTE$^{2-}$, respectively. To this end, extensive quantum-chemical calculations were performed to uncover the nature of the transitions observed in the absorption spectra of the neutral PTTE, monoanionic PTTE$^{-}$ and dianionic PTTE$^{2-}$. First, vertical excitation energies and oscillator strengths were calculated with post-Hartree–Fock methods and methods based on density functional theory. The regular time-dependent density functional theory calculations exhibited the expected trend of increasing excitation energies with higher amount of exact exchange, whereas the results from simplified density functional theory were less systematic. Compared with the post-Hartree–Fock calculations, CAM-B3LYP turned out as best performing functional for absorption spectrum simulations with both density functional theory based approaches. To properly understand the nature of the experimentally observed absorption bands, the simulation of vibronically-resolved spectra turned out to be indispensable. This was demonstrated for the two lowest energy absorption bands of the anion. Based on calculations of vertical excitation energies and oscillator strengths, one might have expected that for each of the bands there is one distinct electronic state causing the absorption. However, calculations taking into account Franck–Condon and Herzberg–Teller terms show that both bands are composed of absorption from both excited states. Ultimately, this fundamental study may act as a guideline for future investigations and potential applications of the promising perylene ester family and underscores the importance of taking into account vibronic effects for proper assignments of optical absorption spectra.

## Methods

The CV measurements were carried out at room temperature on a Metrohm-Autolab potentiostat PGSTAT with PTTE solved in CH$_2$Cl$_2$ with a concentration of $c = 1\,{\text {mmol/L}}$ referenced to the internal standard Fc/Fc$^+$, with [N(n-Bu)$_4$]PF$_6$ as supporting electrolyte ($c = 0.1\,{\text {mol/L}}$), a Pt working electrode, an Ag/0.01 M AgNO$_3$/MeCN reference electrode, a Pt-wire counter electrode, and at a scan rate of $v = 50\,{\text {V/s}}$. The spectroscopy measurements were carried out under the same conditions using a Varian spectrometer Cary 50 and a Pt-mesh working electrode (50 mesh). All measurements were performed under Ar-atmosphere with dry and degassed solvents.

The molecular structure of the investigated compound was created with GaussView^57^. For computational reasons, the aliphatic side chains were replaced by hydrogen atoms, which is expected to have a negligible impact on the investigated properties. Initial structure optimizations for the neutral, monoanionic, and dianionic species were performed on the MP2/6-311g(d,p) level of theory as implemented in the Gaussian 16 software suite^58,59^. Subsequent frequency analysis showed that the optimized structures are minima on the potential energy surface. These structures were the common starting point for the excited state calculations based on either DFT or post-Hartree–Fock methods.

The regular time-dependent DFT (TD-DFT) calculations for the first 30 excited states were also realized with the Gaussian 16 software suite^59^. In our benchmark, we included the BLYP^60,61^ functional as a representative of the generalized gradient approximation, the three global hybrid functionals TPSSh^62,63^, B3LYP^64^ and PBE0^65,66^ with increasing amounts of exact exchange, and the range-separated CAM-B3LYP^67^ functional. In these calculations, the aug-cc-pVDZ^68^ basis set was employed, which was also used in the post-Hartree–Fock and semiempirical calculations to facilitate comparisons between the different methods. The simplified TD-DFT (sTD-DFT) calculations^69–71^ of all excited states up to $15\,{\text {eV}}$ were performed with the same functionals as for regular TD-DFT calculations and were realized with the Orca program^72,73^. Turbomole^74,75^ was employed for the calculation of the first 20 excited states with the RI-CC2^76,77^ and RI-ADC(2)^78,79^ methods together with appropriate basis sets for the RI approximation^80^. Apart from the regular calculations, we have also utilized the SCS and SOS variants of these methods^81–83^. In all post-Hartree–Fock calculations, core orbitals were frozen and a tightened SCF threshold of $10^{-8} \, {\mathrm {Hartree}}$ was used.

To obtain spectra comparable with experiments down to ca. $260 \, {\mathrm {nm}}$, 80 excited states for each species were calculated with the CAM-B3LYP functional and each transition was convoluted with a Gaussian function with a full width at half maximum of ca. $0.25\,{\text {eV}}$. To understand the nature of the electronic transitions that are responsible for light absorption, natural transition orbitals (NTOs) were calculated with the TheoDORE package and the results were visualized using Jmol^56,84–86^. For the first excited state of the neutral molecule and the first two excited states of the monoanion, vibronically resolved spectra including both Franck–Condon and Herzberg–Teller terms were simulated with Gaussian 16^87^. For technical reasons, these calculations used the somewhat smaller 6-311+g(d,p)^58^ basis set and only CAM-B3LYP as functional. The calculations employed the time-independent formulation with combinations of up to eight simultaneously excited modes and a maximum number of $10^{10}$ integrals per class. The vibronic transitions were broadened with a half-width at half-maximum of $400\,{\text {cm}}^{-1}$. With these settings and for all three states, the convergence of the simulated spectra towards the analytical limit reached at least $97.5\%$.

## Supplementary Information

Supplementary Information.

Supplementary Video 1.

Supplementary Video 2.

Supplementary Video 3.
