# Supplementary material for: Unraveling the electrochemical and spectroscopic properties of neutral and negatively charged perylene tetraethylesters
Source: Sci Rep. 2021 Aug 9;11:16097. doi: 10.1038/s41598-021-95551-0 (PMC8352899; doi:10.1038/s41598-021-95551-0)
Supplement: Supplementary file 1 — Supplementary Information. [file 41598_2021_95551_MOESM1_ESM.pdf]

Unraveling the Electrochemical and Spectroscopic Properties of Neutral and Negatively Charged Perylene Tetraethylesters - Supporting Information

Christian Wiebeler[1,2,3,*,†], Joachim Vollbrecht[4,5,*,†,‡], Adam Neuba[4], Heinz-Siegfried Kitzerow[4,5], and Stefan Schumacher[5,6]

[1]Institut für Analytische Chemie, Leipzig University, 04103 Leipzig, Germany
[2]Wilhelm-Ostwald-Institut für Physikalische und Theoretische Chemie, Leipzig University, 04103 Leipzig, Germany
[3]Leibniz Institute of Surface Engineering, 04318 Leipzig, Germany
[4]Department of Chemistry, University of Paderborn, 33098 Paderborn, Germany
[5]Center for Optoelectronics and Photonics Paderborn, University of Paderborn, 33098 Paderborn, Germany
[6]Department of Physics, University of Paderborn, 33098 Paderborn, Germany
[*]christian.wiebeler@uni-leipzig.de, vollbrecht@uni-potsdam.de
[†]these authors contributed equally to this work
[‡]Current address: Institute of Physics and Astronomy, University of Potsdam, 14476 Potsdam-Golm, Germany

ABSTRACT

A detailed investigation of the energy levels of perylene-3,4,9,10-tetracarboxylic tetraethylester as a representative compound for the whole family of perylene esters was performed. It was revealed via electrochemical measurements that one oxidation and two reductions take place. The bandgaps determined via the electrochemical approach are in good agreement with the optical bandgap obtained from the absorption spectra via a Tauc plot. In addition, absorption spectra in dependence of the electrochemical potential were the basis for extensive quantum-chemical calculations of the neutral, monoanionic, and dianionic molecules. For this purpose, calculations based on density functional theory were compared with Post-Hartree-Fock methods and the CAM-B3LYP functional proved to be the most reliable choice for the calculation of absorption spectra. Furthermore, spectral features found experimentally could be reproduced with vibronic calculations and allowed to understand their origins. In particular, the two lowest energy absorption bands of the anion are not caused by absorption of two distinct electronic states, which might have been expected from vertical excitation calculations, but both states exhibit a strong vibronic progression resulting in contributions to both bands.

1 Further Results from Vertical Excited State Calculations

Table S1. Excitation energies for states with oscillator strengths larger than 0.02 for the neutral, monoanionic, and dianionic species of PTTE obtained *via* RI-CC2/aug-cc-pVDZ calculations with and without spin-scaling based on MP2/6-311G(d,p) optimized structures.

**Exp.**			**SOS**			**CC2**			**SCS**	
**ΔE (eV)**	**λ (nm)**	**ΔE (eV)**	**λ (nm)**	**f**	**ΔE (eV)**	**λ (nm)**	**f**	**ΔE (eV)**	**λ (nm)**	**f**
**Neutral**										
2.76	450	3.04	408	0.6468	2.74	452	0.6086	2.94	422	0.6364
**Anion**										
1.28	970	1.66	748	0.1489	1.57	791	0.0611	1.64	757	0.1419
1.44	860	1.69	732	0.0685	1.59	778	0.1274	1.65	750	0.0664
1.97	630	2.40	516	0.6145	2.29	541	0.5984	2.37	524	0.6116
**Dianion**										
-	-	1.95	636	0.2083	1.76	704	0.1746	1.89	656	0.1980
-	-	2.63	471	0.8233	2.43	510	0.8300	2.57	483	0.8280

The results from RI-CC2 and its spin-scaled variants are shown in Tab. S1. The three methods agree in so far that the lowest-lying excited state of the neutral molecule is bright with an oscillator strength slightly above 0.60. However, their

energies differ more markedly, *i.e.* unscaled RI-CC2 with a wavelength of 452 nm lies in the experimental absorption band, whereas its SCS- and SOS-variants are blue shifted by 0.20 eV and 0.30 eV, respectively. For the monoanion, all three methods yield two relatively dark low-lying states, which are most likely the origin of the absorption in the infrared part of the spectrum found experimentally. Another absorption feature that is connected to the formation of the monoanionic species is located around 630 nm. This is caused by a bright state with similar oscillator strength as the bright state of the neutral molecule and it is the fourth excited state in the RI-CC2 calculations, whereas it is the third one in case of the spin-scaled variants. Finally and based on the present calculations, it is expected that the dianion will exhibit absorption at two distinguished positions: Slightly higher in energy than the two lowest-lying states of the monoanion and in-between the prominent absorption bands of the neutral and the singly charged species. For each method and in both regions, an excited state with an oscillator strength larger than 0.10 is found, but there are also several nearly dark states close to them. Therefore, with increasing negative charge of the molecule more and more excited states have to be determined to cover a certain spectral range.

Table S2. Excitation energies for states with oscillator strengths larger than 0.02 for the neutral, monoanionic, and dianionic species of PTTE obtained *via* RI-ADC(2)/aug-cc-pVDZ calculations with and without spin-scaling based on MP2/6-311G(d,p) optimized structures.

Exp.			SOS			ADC(2)			SCS	
ΔE (eV)	λ (nm)	ΔE (eV)	λ (nm)	f	ΔE (eV)	λ (nm)	f	ΔE (eV)	λ (nm)	f
**Neutral**										
2.76	450	3.01	412	0.7327	2.71	457	0.6426	2.91	426	0.7064
**Anion**										
1.28	970	1.64	758	0.1950	1.50	824	0.0594	1.61	769	0.1910
1.44	860	1.66	747	0.0691	1.55	799	0.1811	1.61	769	0.0662
1.97	630	2.44	508	0.4956	2.31	537	0.4645	2.40	516	0.4879
**Dianion**										
-	-	1.90	652	0.2462	1.69	733	0.2018	1.84	675	0.2327
-	-	2.62	473	0.8374	2.38	520	0.7760	2.55	487	0.8221

Qualitatively the same also holds for the RI-ADC(2) calculations, see Tab. S2. More in detail, the excitation energies from these calculations are slightly red shifted relative to their RI-CC2 counterparts. However, these differences are smaller than the ones between the different variants of RI-CC2 and RI-ADC(2). Furthermore, the bright state of the monoanion is now the third excited state for all ADC(2) variants employed.

Table S3. Excitation energies for states with oscillator strengths larger than 0.02 for the neutral, monoanionic, and dianionic species of PTTE obtained *via* regular TD-DFT/aug-cc-pVDZ calculations employing the functionals BLYP, TPSSh and B3LYP on MP2/6-311G(d,p) optimized structures.

Exp.			BLYP			TPSSh			B3LYP	
ΔE (eV)	λ (nm)	ΔE (eV)	λ (nm)	f	ΔE (eV)	λ (nm)	f	ΔE (eV)	λ (nm)	f
**Neutral**										
2.76	450	2.19	566	0.3976	2.39	518	0.4719	2.46	505	0.5029
**Anion**										
1.28	970	1.30	952	0.0296	1.42	871	0.0372	1.48	840	0.0421
1.44	860	1.45	855	0.0338	1.53	810	0.0564	1.56	794	0.0610
1.97	630	2.08	597	0.4554	2.19[1]	566[1]	0.4554[1]	2.21	560	0.5023
**Dianion**										
-	-	1.42	871	0.0750	1.59	781	0.0945	1.66	747	0.1123
-	-	2.16	574	0.5313	2.35	529	0.6160	2.39	519	0.6429

In Tab. S3, the results from regular linear-response TD-DFT calculations for the GGA functional BLYP and the two hybrid functionals TPSSh and B3LYP are shown. For the neutral molecule, the first excited state is always bright, but with increasing amount of exact exchange both excitation energy and oscillator strength increase. Also the first two excited states of the monoanion are similar in oscillator strength relative to RI-ADC(2), but their difference in energy increases with decreasing amount of exact exchange. Furthermore, between these two states and the next bright state there are either two additional states

[1] There is an additional non-dark state at 2.17 eV (571 nm) with $f = 0.0563$.

(BLYP and TPSSh) or one additional state (B3LYP) with low oscillator strengths. In case of the dianion and with the exception of BLYP, the first states are once again found slightly higher in energy than the two dark states of the monoanion. Furthermore, the brightest state of this species is now relatively close to the bright state of the neutral molecule and their difference increases with increasing amount of exact exchange. The findings and trends reported for the functionals BLYP, TPSSh and B3LYP also hold for PBE0 and the long-range separated CAM-B3LYP functional, see Tab. S4.

Table S4. Excitation energies for states with oscillator strengths larger than 0.02 for the neutral, monoanionic, and dianionic species of PTTE obtained *via* regular TD-DFT/aug-cc-pVDZ calculations employing the functionals PBE0 and CAM-B3LYP on MP2/6-311G(d,p) optimized structures. In case of the latter functional, also results from calculations with a polarizable continuum model (PCM) are reported.

**Exp.**			**PBE0**			**CAM-B3LYP**			**+PCM**	
**ΔE (eV)**	**λ (nm)**	**ΔE (eV)**	**λ (nm)**	**f**	**ΔE (eV)**	**λ (nm)**	**f**	**ΔE (eV)**	**λ (nm)**	**f**
**Neutral**										
2.76	450	2.53	490	0.5261	2.76	449	0.6189	2.68	463	0.7879
**Anion**										
1.28	970	1.52	817	0.0448	1.64	757	0.0591	1.58	785	0.0821
1.44	860	1.58	784	0.0785	1.66	748	0.1315	1.64	755	0.2284
1.97	630	2.24	554	0.5215	2.27	545	0.5395	2.17	571	0.6336
**Dianion**										
-	-	1.72	721	0.1203	1.90	652	0.1722	1.84	675	0.2198
-	-	2.45	506	0.6167	2.61	475	0.7990	2.50	495	1.0411

Table S5. Excitation energies for states with oscillator strengths larger than 0.02 for the neutral, monoanionic, and dianionic species of PTTE obtained *via* simplified TD-DFT/aug-cc-pVDZ calculations employing the functionals BLYP, TPSSh and B3LYP on MP2/6-311G(d,p) optimized structures.

**Exp.**			**BLYP**			**TPSSh**			**B3LYP**	
**ΔE (eV)**	**λ (nm)**	**ΔE (eV)**	**λ (nm)**	**f**	**ΔE (eV)**	**λ (nm)**	**f**	**ΔE (eV)**	**λ (nm)**	**f**
**Neutral**										
2.76	450	-	-	-	2.29	542	0.4750	2.19	567	0.4558
**Anion**										
1.28	970	1.28	971	0.0313	1.48	836	0.0415	1.38	898	0.0412
1.44	860	1.43	864	0.0279	1.63	762	0.0373	1.50	827	0.0338
1.97	630	2.06	601	0.4480	2.31[2]	537[2]	0.4868[2]	2.18	570	0.4922
**Dianion**										
-	-	1.31	948	0.0698	1.51	823	0.0939	1.41	881	0.0974
-	-	2.06	602	0.4967	2.27	545	0.5896	2.13[3]	582[3]	0.2958[3]

[2] There is an additional non-dark state at 2.28 eV (544 nm) and $f = 0.0575$.
[3] There is another non-dark state close to it with 2.14 eV (579 nm) and $f = 0.2653$.

Table S6. Excitation energies for states with oscillator strengths larger than 0.02 for the neutral, monoanionic, and dianionic species of PTTE obtained *via* simplified TD-DFT/aug-cc-pVDZ calculations employing the functionals PBE0 and CAM-B3LYP (CAM) on MP2/6-311G(d,p) optimized structures.

**Exp.**			**PBE0**			**CAM**	
**ΔE (eV)**	**λ (nm)**	**ΔE (eV)**	**λ (nm)**	**f**	**ΔE (eV)**	**λ (nm)**	**f**
**Neutral**							
2.76	450	2.17	572	0.4523	2.37	524	0.5863
**Anion**							
1.28	970	1.35	918	0.0408	1.50	827	0.0691
1.44	860	1.47	845	0.0349	1.64	755	0.0622
1.97	630	2.16	575	0.4993	2.23	556	0.6124
**Dianion**							
-	-	1.37	902	0.0869	1.53	810	0.1263
-	-	2.10	589	0.5514	2.14	579	0.6468

2 NTOs for the Charged Species

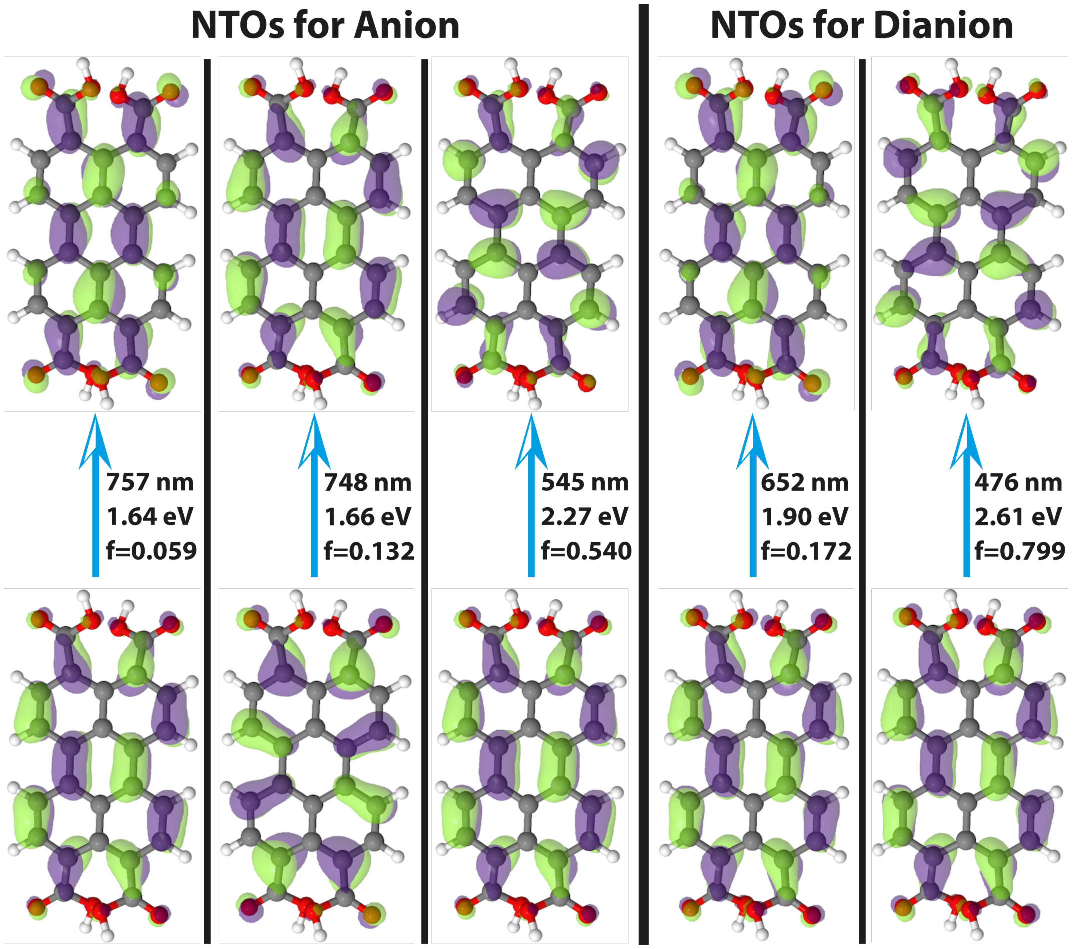

Figure S1. Natural transition orbitals (NTO) of the monoanion (left) for S_1, S_2, and S_3 as well as the NTOs of the dianion (right) for S_1 and S_9. The excited state calculations employed CAM-B3LYP/aug-cc-pVDZ for MP2/6-311G(d,p) optimized structures.

3 Further Results Related to the Vibronic Calculations

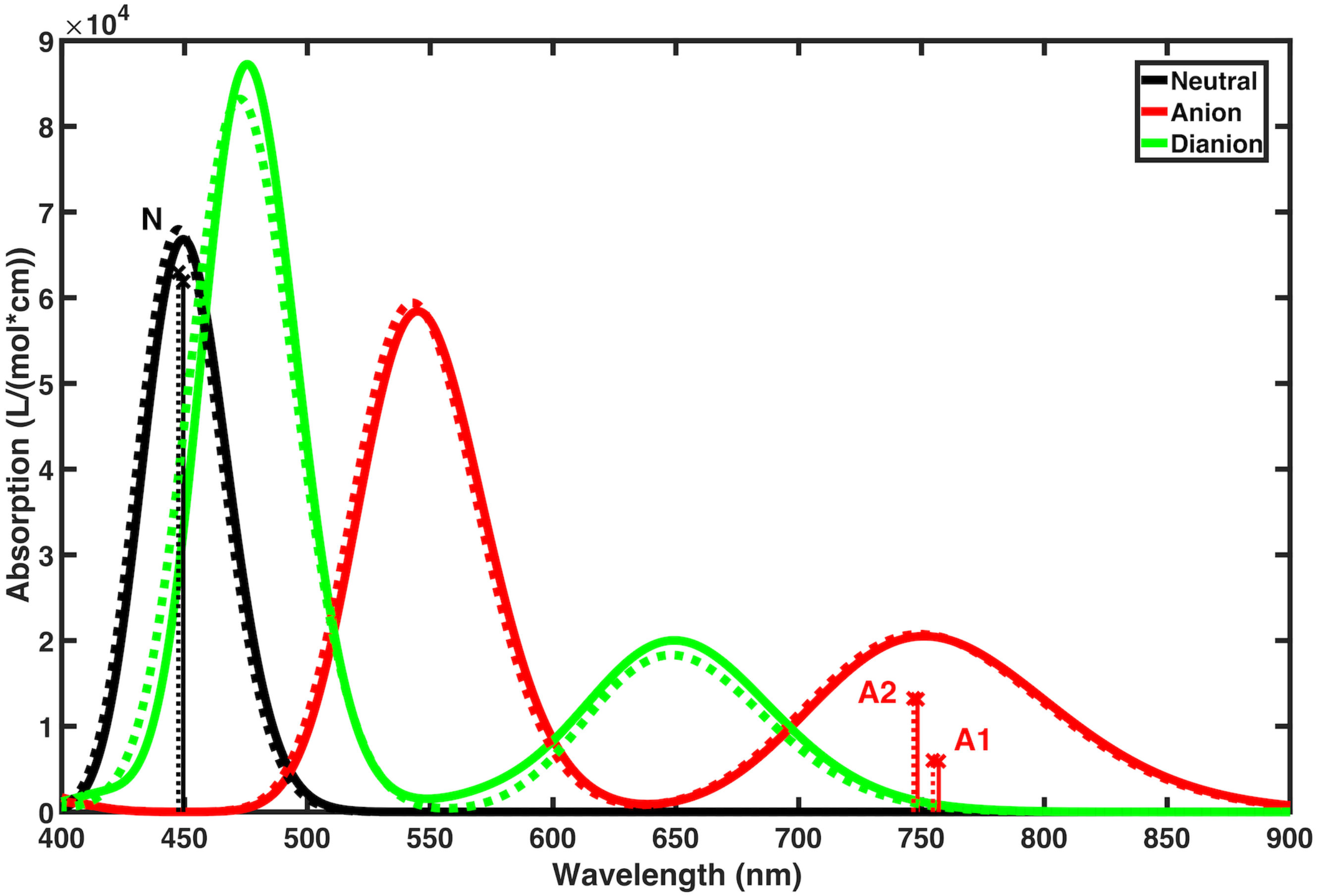

Figure S2. Absorption spectra of the neutral (black) molecule as well as of the singly (red) and doubly (green) negatively charged ions. The excited state calculations employed the CAM-B3LYP functional either combined with the aug-cc-pVDZ (solid) or the 6-311+G(d,p) (dashed) basis and were realized for MP2/6-311G(d,p) optimized structures. The sticks indicate the transitions, for which the vibronic structures were simulated.

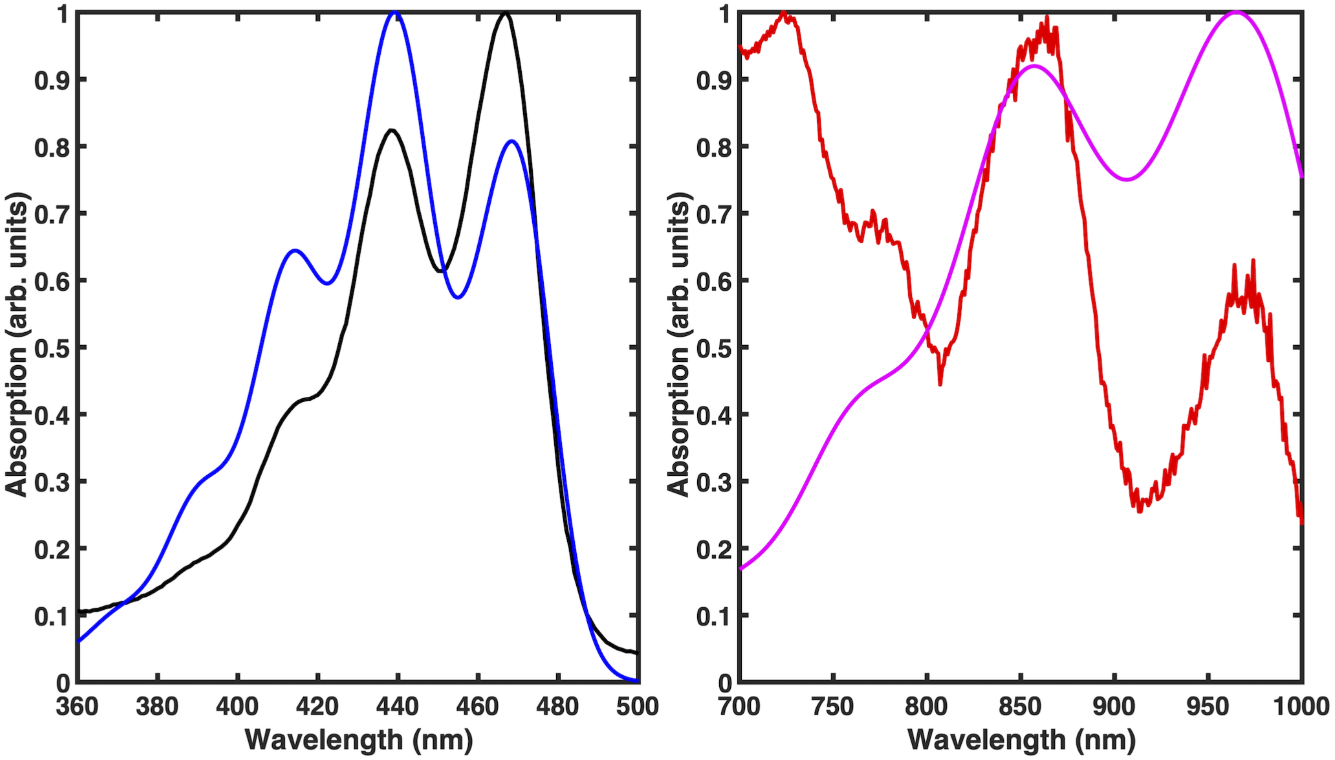

Figure S3. Left: Lowest energy absorption band of the neutral molecule and simulated vibronically resolved absorption spectrum for state N. No shift was applied to the simulated spectra. Right: Absorption in the red to near infrared region found experimentally and simulated vibronically resolved spectrum as sum of absorption from states A1 and A2. The simulated spectra are red-shifted by 1500 cm^{-1} for better comparability.

As can be seen in Fig. S3, the position of the simulated absorption band for the neutral molecule matches quite well to experiment. One of the reasons might be that the CAM-B3LYP functional yields excitation energies that are only slightly higher than from Post-Hartree-Fock methods, *i.e.* 0.02 eV relative to RI-CC2 and 0.05 eV relative to RI-ADC(2). Furthermore, including the solvent with a polarizable continuum model leads to a red shift of 0.08 eV for the vertical calculations, see Tab. S4. Therefore, employing one of the mentioned Post-Hartree-Fock methods for the electronic structure and/or including the solvent would be expected to disimprove the agreement by red-shifting the absorption.

In contrast to this, the absolute positions of the simulated absorption bands from A1 and A2 have to be red-shifted by *ca.* 0.19 eV to achieve coincidence with the positions of the maxima from experiment. A smaller part of this shift might be recovered by employing PCM, which leads to a lowering in vertical excitation energies by 0.06 eV and 0.02 eV for A1 and A2, respectively. In addition, excitation energies from CAM-B3LYP are 0.07 eV higher for both transitions relative to RI-CC2 and relative to RI-ADC(2) by 0.14 eV 0.11 eV for A1 and A2, respectively. Owing to this, the agreement might be improved by employing both a solvent model and a Post-Hartree-Fock method.

4 Analysis of Normal Modes Contributing to Vibronic Progression

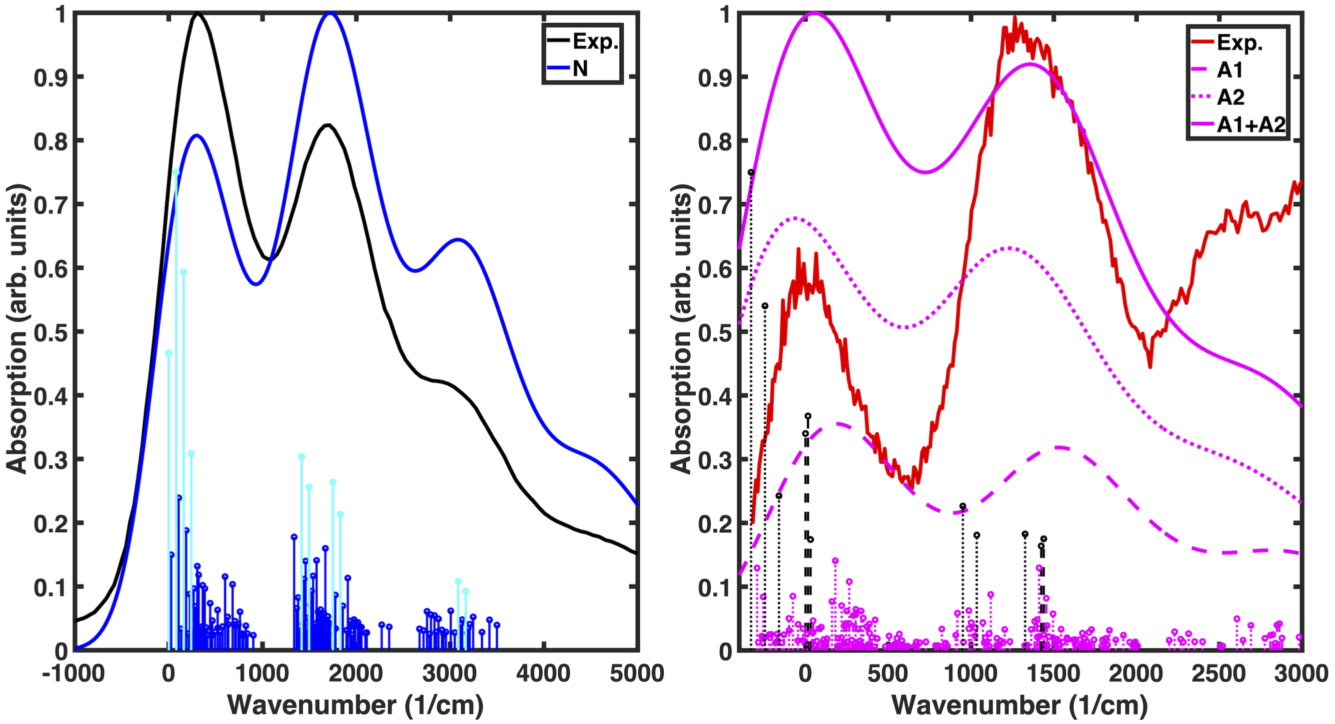

Figure S4. Left: Lowest energy absorption band of the neutral molecule and simulated vibronically resolved absorption spectrum for state N. The energies, by which the spectra are shifted, were set to 21115 cm^{-1} and 21053 cm^{-1} for the experimental and simulated spectra, respectively. Right: Absorption in the red to near infrared region found experimentally and simulated vibronically resolved absorption spectra from states A1 and A2. The energies, by which the spectra are shifted, were set to 10309 cm^{-1} and 11809 cm^{-1} for the experimental and simulated spectra, respectively. This choice of reference energies is slightly different to the one in the manuscript, but taking the 0-0 transition energies of N and A1 as reference for the calculated spectra instead of the first absorption maximum is more appropriate for the discussion of vibronic progression. The references for the experimental spectra were then chosen in such a way that the position of the lowest energy absorption maximum coincides with the simulation. The sticks represent positions and relative intensities of sufficiently strong vibronic transitions and the ones that are discussed in the text are highlighted in cyan and black for the neutral molecule and the anion, respectively.

To further analyze the simulated vibronically resolved absorption spectra, we visualized the individual vibronic transitions in Fig. S4. For the neutral molecule, we find three groups of these transitions causing the first three absorption maxima. The strongest transitions in the first group are the 0-0 transition and transitions involving excitation of mode 5 with one, two or three quanta. This mode corresponds to a tilting movement between the left and right side of the molecule and is illustrated in Fig. S5. The strongest transition in the second group is caused by excitation of mode 90, which is mainly an expansion and contraction of the central benzene unit. Mode 110 is excited in the second strongest transition. This mode is mainly composed of stretching movements of the carbon atoms along the horizontal bonds. The remaining two transitions in this group additionally involve excitation of mode 5. In the third group, the two strongest transitions are composed of simultaneous excitations of modes 90 and 110 either without or with mode 5. Overall, mode 5 is responsible for the dispersion within one group, whereas modes 90 and 110 are leading to the distinct groups of vibronic transitions.

The vibronically resolved absorption spectra for A1 and A2 exhibit a similar grouping as the neutral molecule. In case of the former, the strongest transitions in the first group are again the 0-0 transition as well as excitation of a tilting vibration similar to mode 5 of the neutral molecule. However, this vibration is now lower in energy and the first mode, see Fig. S6. Furthermore, only excitations with one or two quanta exhibit strong intensity. Mode 98 is a similar stretching vibration as mode 110 from the neutral molecule. Excitation of this mode either with or without excitation of mode 1 results in the two most intense transitions of the second group. For A1, only two normal modes are sufficient to describe the five most intense transitions and they resemble two modes discussed for the neutral molecule, but are somewhat lower in energy.

In contrast to this, three modes are again involved in the most intense vibronic transitions of A2, see Fig. S7. These

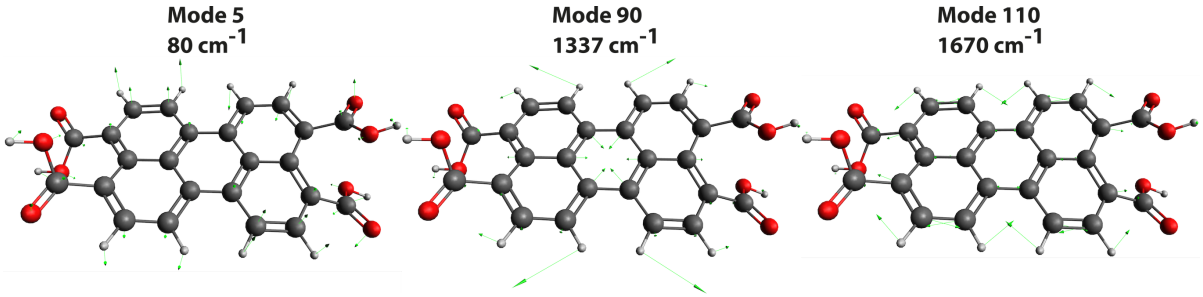

Figure S5. Normal modes for N that are involved in the vibronic transitions highlighted in cyan in Fig. S4. The insets indicate the number of the mode in energetic order and its energy.

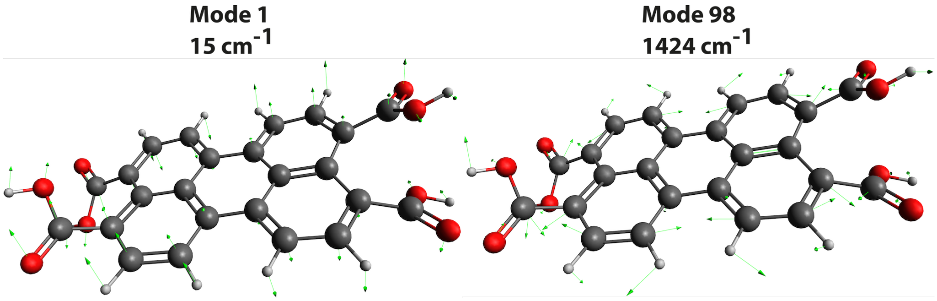

Figure S6. Normal modes for A1 that are involved in the vibronic transitions highlighted in black with dashed sticks in Fig. S4. The insets indicate the number of the mode in energetic order and its energy.

vibrations are quite similar in displacements and energies to their counterparts from the neutral molecule. The first group consists of the 0-0 transition and excitation of mode 7 by one or two quanta. The first two transitions in the second group are excitations of mode 87 without or with mode 7 and the remaining transition is an excitation of mode 110.

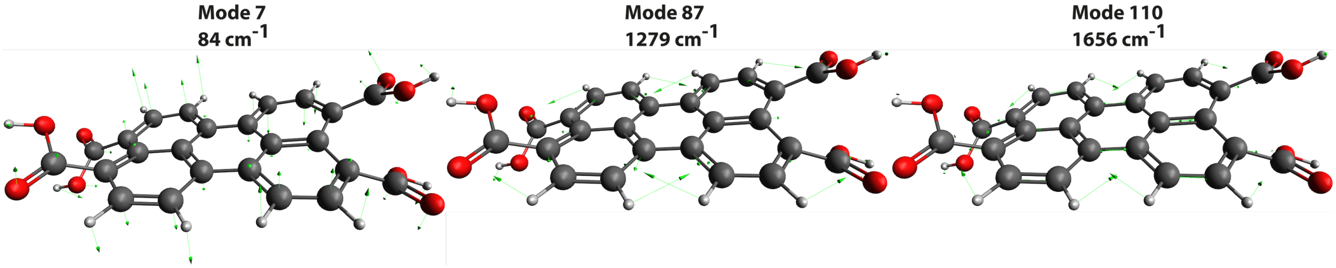

Figure S7. Normal modes for A2 that are involved in the vibronic transitions highlighted in black with dotted sticks in Fig. S4. The insets indicate the number of the mode in energetic order and its energy.

Overall, the vibrational excitations contributing the strongest to the vibronic band shapes can be traced back to three types of modes. In particular, the involved vibrational modes for N and A2 are rather similar, which is further corroborated by the NTOs of these transitions, see Figs. 3 and S1: The electron is excited from a π orbital with contributions along bonds involved in the contraction and expansion of the central benzene ring into a π orbital that is aligned along the long axis with contributions along bonds that are part of the C-C stretching vibration. Contrary to this, both NTOs of A1 are aligned along the long axis, but not along the bonds that are involved in the benzene ring contraction/expansion explaining why the latter vibration does not contribute strongly to the vibronic progression of this transition.
